# The Work of the Hospitals Association

**Published:** 1889-10-26

**Authors:** 

**Affiliations:** President, Senior Physician to St. Thomas's Hospital


### The Work of the Hospitals Association.

By Dr. Bristowe, F.R.S., President, Senior Physician to St. Thomas's Hospital.

The Hospitals Association, which has now been in existence nearly six years, was born of a conference on hospital administration, initialled and organised by Mr. Burdett, and held under the auspices of the Social Science Association in July, 1883. The conference—which met under the presidency on the first day of Sir T. Fowell Buxton, Bart., and of Mr. Francis S. Powell on the second, and was attended by representatives of many metropolitan and provincial hospitals—came appropriately to an end with the adoption of the following resolution :

"Proposed by Dr. T. Gilbart Smith, seconded by Dr. Edward Seaton, and carried unanimously, 'That the council of the Social Science Association be requested to invite the following attendants at the conference to form themselves into a committee, with power to add to their number, to consider what steps, if any, should be taken to secure combined action among hospitals, and to decide as to future conferences, and to take such other steps as may appear desirable: the Earl of Cork and Orrery, Viscount Powerscourt, Sir Thomas Fowell Buxton, Bart., Dr. (now Sir Henry) Acland, Dr. Bristowe, Mr. Henry C. Burdett, the Rev. Canon Erskine Clarke, Dr. R. Farquharson, M.P., Mr. S. Leigh Gregson, Mr. J. J. Gurney, Mr. Timothy Holmes, Mr. J. B. Lloyd, Mr. Francis S. Powell, and Mr. Joseph White.'

Many others joined the committee, which ultimately brought the claims of such an association more prominently under the notice of the public at an influential meeting held at the Mansion House under the presidency of the Lord Mayor, Mr. Alderman (now Sir Robert) Fowler, Bart., M.P., on the 1st Feb., 1884, when a large number of representatives of metropolitan and provincial hospitals attended to support the formation of the association. After a statement by Major Ross, the chairman of the provincial committee, on the scope and objects of the society, Sir T. F. Buxton moved, Sir Rutherford Alcock seconded, and Mr. Timothy Holmes supported, a resolution to the effect that it is desirable to establish, under the name of The Hospitals Association, an organisation for the consideration and discussion of matters connected with hospital management.

A second resolution, moved by Earl Stanhope, and seconded by Dr. Farquharson, left the preliminary arrangements connected with the establishment of the association, including the appointment of the first council, to the provisional committee, which acted with such promptness that the first meeting of the council was held on the 5th March, 1884.

Nearly six years have elapsed since this meeting, and although it may be allowed that the association has not yet accomplished all that it hoped to accomplish, and although it has had formidable difficulties to contend with, no one who has watched its progress and studied its proceedings can hesitate to acknowledge that it has done much excellent work, and has fully justified its existence.

The aim of the founders of the Hospitals Association, and, indeed, of the association itself during its whole career, has been an ambitious one—namely, to promote the best interests of hospitals and infirmaries, and of all who are engaged in their administration ; and to this end, to invite the co-operation of all persons interested in the work of hospitals, more especially presidents, treasurers, and governors, medical officers, apothecaries, chaplains, architects, stewards, and secretaries, matrons, sisters, and nurses.

Among the objects which would naturally come under the cognisance of such an association may be enumerated—(1) The construction and arrangement of hospitals, comprising the questions of situation, ventilation, cubic space, and drainage ; (2) the domestic management of hospitals, including cleaning, washing, dietary, etc. ; (3) the arrangements as regards the supply of medicines, surgical appliances, and the like; (4) the nursing of patients, and the comfort, welfare, and education of nurses; (5) the arrangements for the spiritual welfare of the hospital inmates; (6) the means of obtaining funds for the carrying on of the work of institutions wholly or in part dependent on annual subscriptions ; (7) the economical management of hospitals; (8) the duties of hospitals in reference to the teaching of medical men and nurses ; (9) questions relating to the classes of patients to be admitted for treatment, and the terms on which patients should be admitted ; and, lastly, any questicn which may arise having relation to the treatment of the sick, such as the advantages or disadvantages of special hospitals and of provident dispensaries, convalescent homes, and the means of transport for the sick and injured. The above list is not exhaustive, but it doubtless comprises the more important matters that a Hospitals Association would naturally regard it as its province to deal with; and it will be admitted that the list is a large and important one.

Now, it is obvious that many of these subjects are not capable of being dealt with usefully, in the first instance, with a large, heterogeneous body, but that they need to be studied and thought over by those who possess special knowledge and experience, in order that they may be placed before the large body in a form capable of easy comprehension by them. Hence the need of the appointment of special or sectional committees for the investigation of special subjects, and for reporting on them. This was foreseen from the beginning, and it was intended that, as far as possible, the different groups of hospital officials and persons directly interested in hospitals should form such committees, partly for mutual information, support, and benefit, but mainly with the object of determining amongst themselves the best methods of dealing practically with those departments of hospital management with which they were individually concerned, and then of bringing their matured conclusions before the association and the public for utilisation.

I may, perhaps, in relation to this branch of the subject matter of my paper point out that it has always been intended that the office of the association should contain a library of books, pamphlets, and reports on all matters (excepting purely medical matters) concerning hospitals ; that it should be a centre for the storage and diffusion of information with respect to hospitals, and for imparting such information to all enquirers ; and that it should be, in a small way, a rallying point and a club for all persons interested in the objects of the association.

I will now briefly consider how far the programme sketched above has been carried out. In the first place, the association has succeeded in bringing together the classes of representative persons among whom it looked for support, and at the present time we have on the council, and among the members, several noblemen who are interested in philanthropic objects and in hospitals, the treasurers and chairmen of the four largest metropolitan hospitals, physicians and surgeons of high eminence, hospital secretaries and architects, matrons or lady superintendents of hospitals and of nursing institutions, and others whose tastes or special work lead them to investigate or relieve the sufferings of the sick poor. I do not pretend that the numbers of our members are so large, or that the different departments of hospital work are so well represented as we could wish them to be, or as they should be. There are many reasons for this. What's everybody's business is no one's business. And there are many hospital officials who are excellent officers and do their own work well, but who, from various causes, do not care to travel out of their immediate grooves, do not recognise the need of association and discussion, are content with things as they are, and are well satisfied to leave the work of reorganisation and improvement, with all the trouble and annoyance connected therewith, to men of greater mental activity and energy. Unfortunately, too, the medical officers of hospitals, generally, whose special hospital work lies in the investigation and treatment of the cases under their care, have little time on their hands for the duties, and take little personal interest (notwithstanding that they deeply concern them) in the subjects engaging the attention of the Hospitals Association. Again, matrons and nurses are not so well represented in the association as they should be. The explanation is partly, no doubt, that their own work leaves them little leisure, that they are not rich in this world's goods, and that even a small annual subscription can be ill afforded. But it may be due in part to the secession of a few of these ladies from the society which took place about two years ago in connection with the ambitious and, as I think ill-advised, project for the registration of nurses, and the quarrel which some among them, on that account, fastened on the association. But notwithstanding these drawbacks and impediments to progress, the association keeps up its numbers, and reckons amongst its members many of the most eminent persons in their several departments of work or walk in life.

Secondly, as already stated, committees for special duties have been from time to time appointed, and such committees exist at the present moment—the most important we have now in existence being the committee to organise and mature a street ambulance, consisting of Sir Sydney Waterlow, Mr. H. L. Bischoffsheim, Mr. Burdett, Mr. P. Michelli, Mr. T. Ryan, and Dr. Steele.

In the third place, periodical meetings have been held regularly every year, and many papers and subjects of great interest have been brought before them and discussed. Of these, the most weighty and beneficent was the paper on "A National Pension Fund for Hospital Officials and Trained Nurses," read before the association by Mr. Burdett on October 12, 1887, and the paper on the conveyance of injured persons to the metropolitan hospitals, read by Mr. Thomas Ryan on the 20th March of the present year; the first of which resulted in the establishment of the National Pension Fund for Nurses, which the munificent donations of Lord Rothschild, Messrs. Hambro, H. Hucks Gibbs, and Junius S. Morgan so materially aided (as well shown in the report of the council presented to the second general meeting yesterday), is already successful beyond the most sanguine hopes, and promising to be a substantial boon to the nursing profession; the latter of which has resulted in the establishment of a powerful committee, to which I have already alluded, which is at present engaged in supplementing the inadequate and unsatisfactory ambulance arrangements of the metropolis by a street ambulance organisation of the most perfect kind.

I have now placed before you briefly an account of the Hospitals Association, a statement of the views and aims of those who founded it, and of those who have been chiefly instrumental in directing its proceedings down to the present time, and a narrative of the labours of the association and their valuable results. I now appeal to you—and through you to the public—to give the association your continued cordial support, and to aid it in the good work which it strives and hopes to achieve. It will, I think, be generally allowed that the objects of the society are unimpeachable, that there is a real need of such a society as ours, in which the varied interests concerning hospital management, and the relation of hospitals to the sick, to the profession, and to the public, may be adequately discussed, and in which new schemes for the benefit of nurses and others, for the benefiting of patients and of the sick poor generally, may be ventilated, and put into practical shape. But if we are to discuss questions adequately, so as to elicit the truth with regard to them, and to be helped in these discussions with the best available knowledge of them, with a view to useful and successful action, it is obvious that those who know most about them should take an important part in the proceedings, and that their opinion and experience should be brought to bear upon them, and have their due influence. It is of the highest importance, therefore, that persons connected with hospitals should connect themselves largely with the Hospitals Association, and that not only should its members include treasurers, governors, chaplains, medical officers, architects, stewards, and secretaries, and matrons, and sisters, and nurses, but that as far as possible all hospitals and infirmaries, and similar institutions, should be adequately represented in it.

Objections have been made to the association, but all the objections I have heard, or seen advanced, are trivial, and more or less unfounded. It has been said that the association is pledged to certain views and certain lines of action with respect to hospital action and provision for the treatment of the sick poor, and that consequently it is useless and a waste of time for those who do not share the dominant views to connect themselves with it. It is true that some among us entertain strong opinions on certain questions, and are prepared to uphold these opinions, and push them with all our might. But how, I should like to know, would the world make progress if there were not vigorous and masterful minds ready and bold to lead or drive the weaker amongst us to victory or defeat? But, as a matter of fact, the Hospitals Association as an association is pledged to nothing but to use its best influence to promote the interests of hospitals, of hospital workers, and of the sick. It has adopted no theories. The association has been regarded as a mere assembly of busybodies, who concern themselves with all other persons' business except their own. This view is clearly untenable, for among its most active workers are men whose time is largely engaged. The association is an association representing hospital interests. Other accusations have been brought against the association, but I need not allude to any more of them. It is sufficient for me to say that if there be any truth in any of them, they will cease to be valid as the association becomes larger and more representative, and when to prevent the possibility of any misrepresentation of hospital interests, it is only necessary that those interests should be well and sufficiently represented in the association.

Before concluding this brief address, I may call the attention of my audience to two or three important matters which the association has long had under its consideration, and which it hopes—in the interests of the charitable and suffering public—to assist in bringing (if it be possible) to a satisfactory issue. The first is the out-patient question, on which so much has been said and written, and about which so many complicated opinions are held. Within the last eighteen months several papers have been read before the association by those entitled to have an opinion on this subject, but who have dealt with it from quite different points of view. The question is undoubtedly one of great difficulty and even perplexity, but it is on the way to solution, and a body well fitted from its constitution to deal with it successfully is the Hospitals Association, in which all interests are represented. The second concerns hospital accounts and expenditure. No one doubts that there are many hospitals in which, notwithstanding the poverty, the expenditure of the subscribers' money is lavish and wasteful, and that such extravagant expenditure should be put a stop to, but that, unfortunately, the accounts of hospitals are so kept that they convey no due information with regard to the details of expenditure, and are such different systems that the comparison between the expenditure of different hospitals is to a great extent impossible. As Mr. Burdett has long held, and urged before the public, hospital accounts should be required to be kept on a similar and intelligible system, and must be so kept if wasteful expenditure is to be checked. This is a matter with which we have dealt, and hope, with the assistance of the hospitals themselves, to deal effectively.

Lastly, I thank the authorities of Charing Cross Hospital for allowing the first meeting of the present session of the Hospitals Association to be held in their board-room. I feel it my duty to call attention to the important and gratifying fact that during the last two sessions the principal meetings of the association have been held habitually, with the kind consent of the governors, in the board-rooms of London hospitals, and that the committee of the association, with the institution it represents, is recognised on terms which we hope, as years progress, will be more and more intimate.

